# Shortening subtrochanteric osteotomy and cup placement at true acetabulum in total hip arthroplasty of Crowe III–IV developmental dysplasia: results of midterm follow-up

**DOI:** 10.1007/s00590-017-2076-8

**Published:** 2017-11-25

**Authors:** Alireza Manafi Rasi, Gholamhossein Kazemian, Mohammad Khak, Reza Zarei

**Affiliations:** 1 Department of Orthopedic and Trauma Surgery, Shahid Beheshti University of Medical Sciences, Tehran, Iran; 2 Department of Orthopedic and Trauma Surgery, Qazvin University of Medical Sciences, Qazvin, Iran

**Keywords:** Developmental dysplasia of hip, Total hip replacement, True acetabulum, Subtrochanteric osteotomy, Cotyloplasty

## Abstract

**Introduction:**

The anatomic abnormalities in developmental dysplasia of hip (DDH) often make total hip replacement (THR) inevitable at a younger age. However, there is no universal gold standard technique of THR for high dislocated dysplastic hips.

**Materials and methods:**

Here we present the outcomes of midterm follow-up after THR in patients diagnosed with DDH Crowe type III and IV hospitalized in a tertiary center in Iran for whom placement of a cup in true acetabulum and selective transverse subtrochanteric osteotomy was performed. Pre- and postoperative Harris Hip Score, leg length discrepancy and postoperative complications were evaluated.

**Results:**

A total of 48 patients with DDH Crowe type III and IV (uni- or bilateral which made 52 hips) were studied. Mean age of patients was 41 years with minimum follow-up ranging from 12 months to 3 years. Mean Harris Hip Score significantly improved from 41.70 preoperatively to 88.1 at last follow-up postoperatively. Leg length discrepancy of less than 2 cm was observed which was well tolerated using shoe lifts. Regarding postoperative complications, two patients had transient peroneal nerve palsy in early postoperative period which recovered within 2 months. No other major complication was encountered.

**Conclusion:**

THR in patients with DDH (Crowe III and IV) with a cup positioned in true acetabulum and transverse subtrochanteric osteotomy is a safe successful procedure.

**Electronic supplementary material:**

The online version of this article (10.1007/s00590-017-2076-8) contains supplementary material, which is available to authorized users.

## Introduction

Developmental dysplasia of hip (DDH) is labeled as one of the utmost widespread developmental illnesses diagnosed among children leading to secondary osteoarthritis of the hip joint [1, 2]. This states a spectrum of developmental abnormalities which hamper the growing hip and leads to altered anatomy of the acetabulum and proximal femur [3]. Dysplastic hips have one single pathophysiological feature in common; i.e., the anatomic abnormalities raise the contact stress leading to osteoarthritis [4]. Hence, this severe osteoarthritis secondary to DDH often makes total hip replacement (THR) inevitable at a younger age [5, 6]. Although the pathology at the acetabular side of the joint is more prominent, the femoral head may be deformed and high riding with hypoplastic proximal femur.

However, there is no universal gold standard technique of THR for high dislocated dysplastic hips and treatment of each patient should be individualized. Due to some certain characteristics of these patients such as young age as well as anatomic abnormalities of the hip, the complication rate after THR in DDH patients is higher than normal population [2–6]. The new center of hip rotation after cup placement alters hip biomechanics, leg length and femoral reconstruction. Hence, in order to achieve successful acetabular reconstruction during THR in DDH patients, knowing the position of acetabulum is mandatory. Purpose of acetabulum reconstruction is insertion of acetabular component in the true acetabulum for its biomechanical advantages. Crowe classification is the most frequent way of categorizing hip dysplasia [7] (Table 1).

**Table 1:** Crowe classification of adult developmental dysplasia of the hip according to the extent of the underlying subluxation on AP X-ray of the pelvis [7]

Crowe grade	Dislocation	Description
Grade 1	< 50% subluxation	Femur and acetabulum show minimal abnormal development
Grade 2	50–70% subluxation	The acetabulum shows abnormal development, femoral head articulates with false acetabulum, which partially covers the true acetabulum
Grade 3	70–100% subluxation	The acetabulum is developed without a roof
Grade 4	> 100% subluxation (luxation)	The acetabulum is deficient but remains recognizable

Once we are set to select one appropriate treatment model for reconstruction of acetabulum, we are required to bring pros and cons as well as the rate of bone deformity involved to a meticulous consideration. Pros of biomechanical and practical reconstruction of acetabulum and cons of the urgent need for sufficient coverage of implant in acetabulum must be balanced out. Shallow socket with bone deficiency at anterior, lateral and superior aspects of acetabulum is considered as one of vital changes in dysplastic hips. In dislocated hips, the femoral head articulates with iliac bone and results in false acetabulum formation. Force vector direction of abductor muscles that is usually vertical changes to horizontal, and they become shorten and contracted. Despite the advanced methods and the available implants, THR in Crowe grade III and IV DDH is a challenging surgery. The most important part of the treatment is selection of the right technique. Unfortunately, there is no consensus on surgical planning in these patients. The success of operation hangs on the severity of illness.

However, in this study the outcome of 12- 36-month long follow-up after THR in patients diagnosed with DDH Crowe type III and IV hospitalized in a tertiary center in Iran over 2013–2015 in whom placement of a cup in true acetabulum and selective transverse subtrochanteric osteotomy was performed is presented.

## Materials and methods

Study protocol was in accordance with the Declaration of Helsinki for human research and approved by Shahid Beheshti University of Medical Sciences Ethic committee; informed consent was obtained from the patients. In this study, 48 patients with DDH Crowe type III and IV (uni- or bilateral which made 52 hips) who underwent THR between May 2013 and April 2015 in a tertiary referral center in Iran were studied. THR indications included severe pain or functional impairment with difficulty in performing daily living activity and walking (or both). All patients were evaluated clinically and radiographically before surgery. For each cases, according to Harris Hip Score [8] pain and grade of disability were assessed in terms of limitation of hip range of motion, limb length discrepancy and restriction on walking and in doing daily activities. Preoperative shortening was radiographically measured between tear drop on pelvis and lesser trochanter on femur. The patients were followed up clinically and radiographically at 2 weeks, 3, 6 and 12 months after surgery and yearly thereafter.

**Fig. 1:**
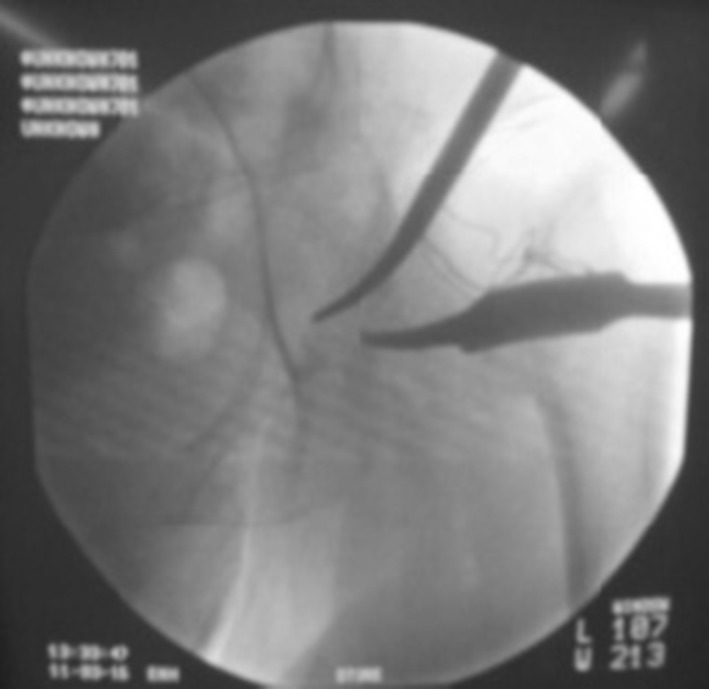
Localizing true acetabulum with the help of image intensifier

**Fig. 2:**
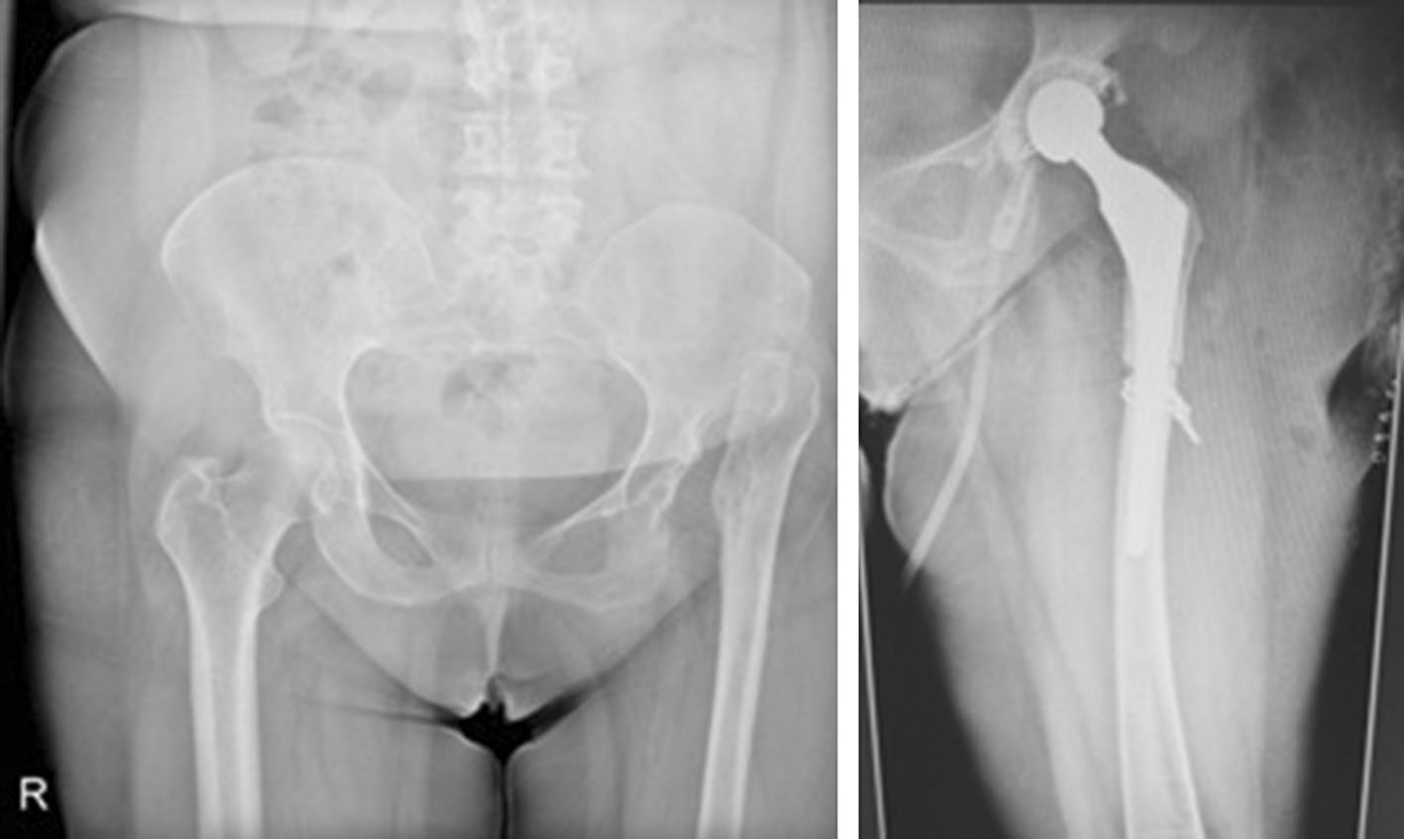
In two cases, we used cemented cup for THR. Distal fitting stem was also used

**Fig. 3:**
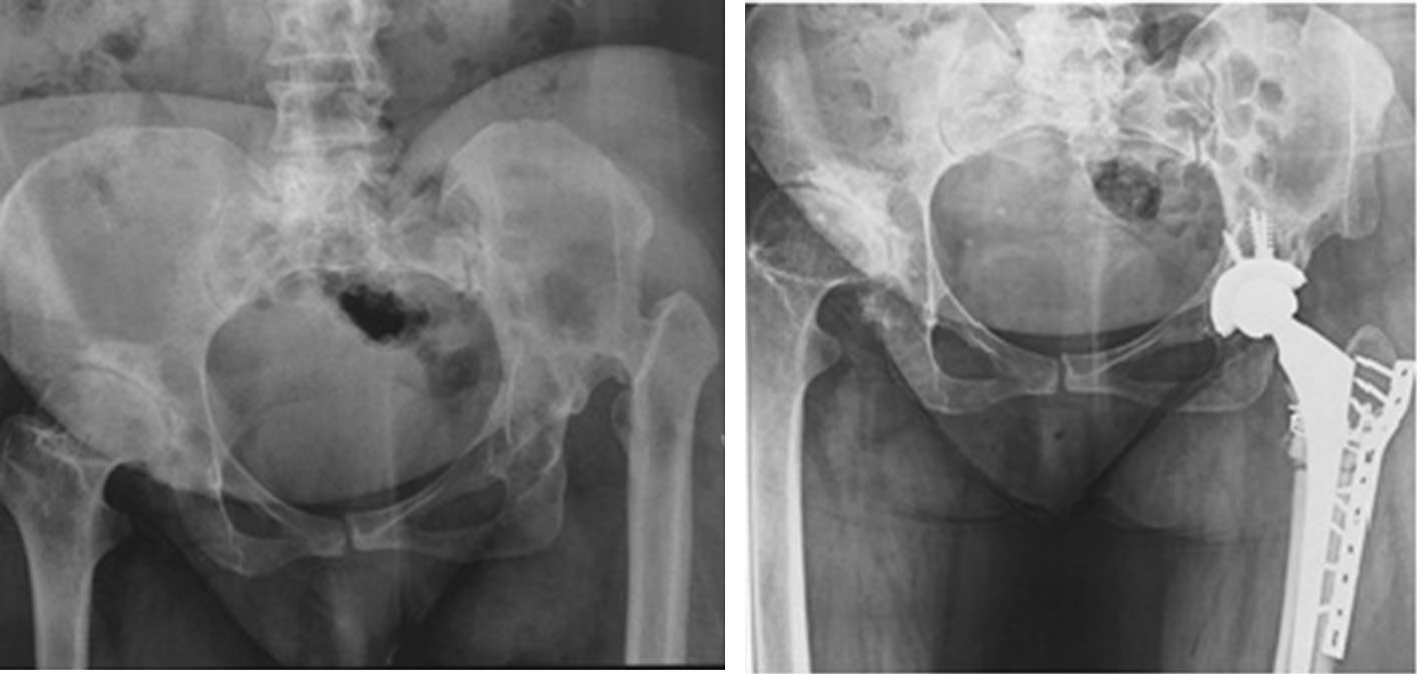
High-riding DDH which underwent THR with cup placement in true acetabulum using multiple screws; transverse subtrochanteric osteotomy was performed and fixed with a plate

All the surgeries were conducted by a single surgeon (AMR) in lateral position using Harding approach. Soft tissue dissection is conducted carefully keeping in mind that neurovascular bundle may not be present at normal anatomic place. During the surgery, gluteus minimus is released from the top of anterior tuberosity. The femoral neck is resected at around 1 cm proximal to lesser trochanter. Pushing down toward capsule and by direct palpation, the correct position of the true acetabulum is located. Although the precise position of acetabular component is well determined in preoperative radiographs, the whole process of finding the true acetabulum is monitored by image intensifier (Fig. 1). After sufficient soft tissue dissection, the acetabulum is deepened and enlarged gradually with serial reaming. The first ream is done in a posterior-directed fashion with reamer size 36. Cotyloplasty (medialization of the anatomic joint) is also carried out. Then, we keep reaming at the desired angle of abduction and anteversion till anterior and posterior wall appear in order to fix a cementless component with two to three screws. It is reamed to size 39–40, and the cup (size 40–44) is applied. However, a cemented cup was applied for two patients (Fig. 2). After capturing a proportional press fit, the cup is tightened with long screws (at least one screw per dome) (Fig. 3).

**Fig. 4:**
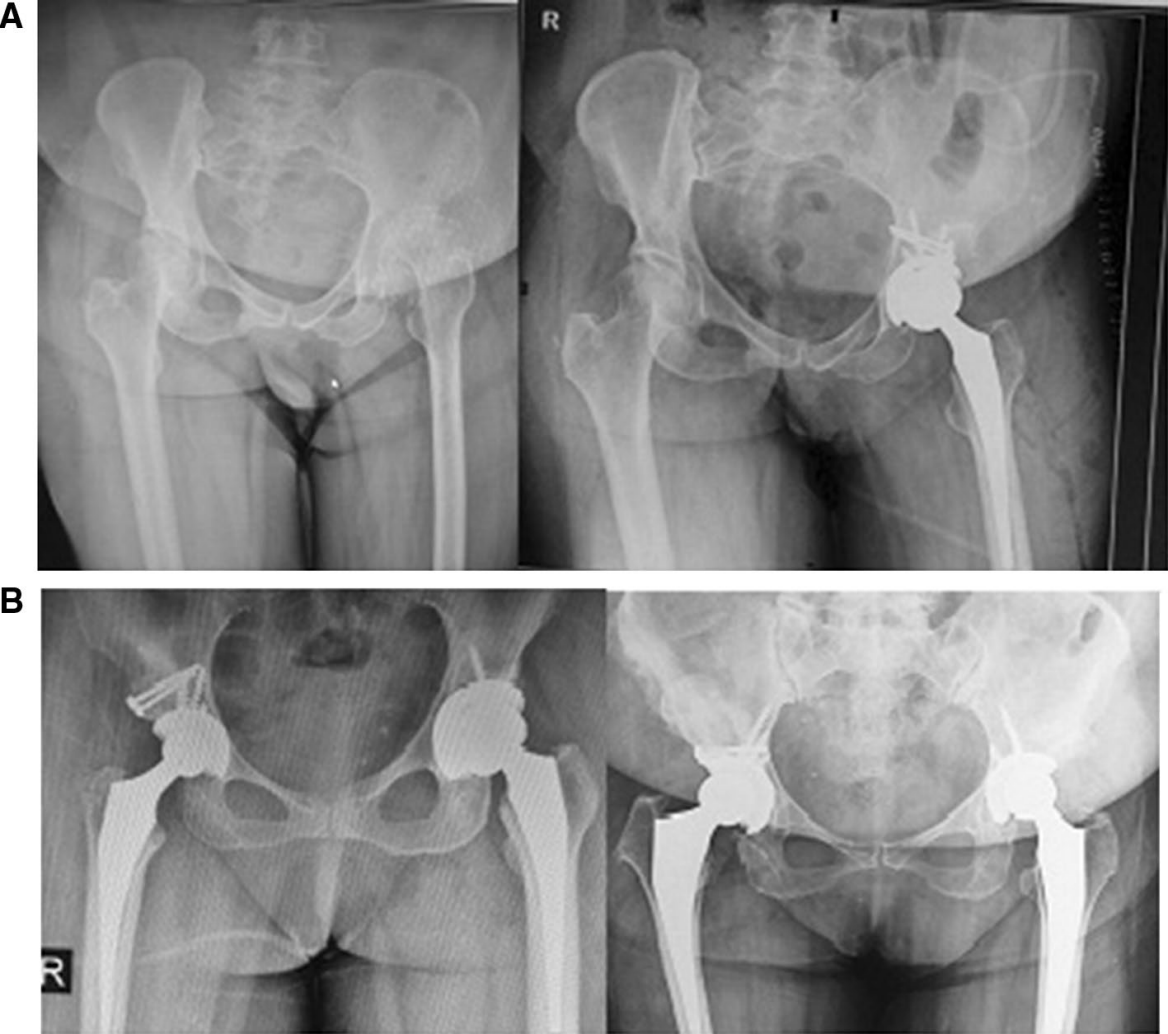
Use of autograft in cases with over than 30% of cup uncovered (**a**). Postoperative radiographs of two other patients after graft union (**b**)

**Fig. 5:**
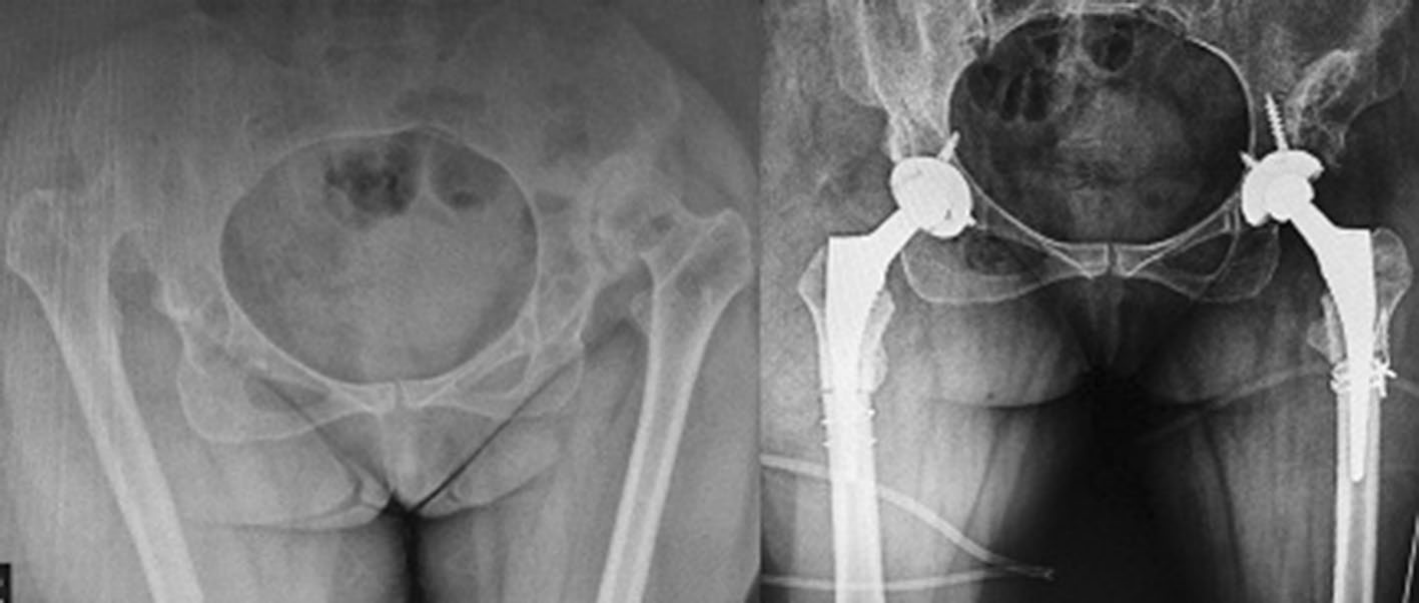
Bilateral DDH undergone THR with small cup placed in true acetabulum and transverse subtrochanteric osteotomy

If the acetabular roof is deficient with more than 30% of the cup uncovered, we would reinforce the superior section with placement of cortico-cancellous structural bone graft from femoral head. This was done in 5 acetabula (Fig. 4a, b). The acetabular component is then impacted and fixed with screws. In some cases, the acetabulum was very small and reconstruction was possible only with smaller cups (Fig. 5).

**Fig. 6:**
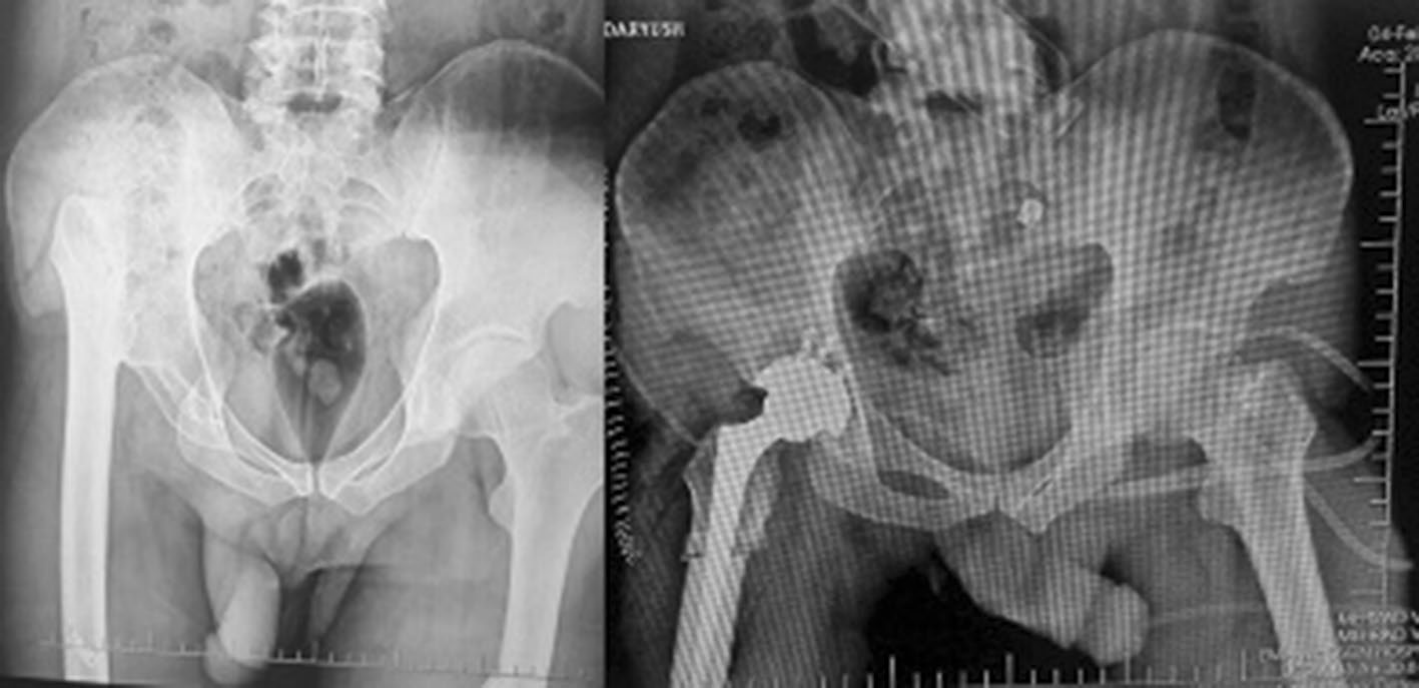
High-riding DDH undergone THR with cup placement in true acetabulum, fixed with three screws and transverse subtrochanteric osteotomy

For femoral components, a primary reaming is done at first. Bringing down of femoral head to the level of the true acetabulum is impossible in some hips after proximal dislocation because of soft tissue contracture. In such cases, a transverse subtrochanteric osteotomy is performed (Fig. 6). Measurement of subtrochanteric osteotomy is done intraoperatively. After reduction in the hip joint, we stretch the leg (with moderate tension) at a conventional length and mark the proximal and distal overlap and have it transversely excised. The medullary canal is reamed progressively. Finally, we lay the femur trial over the proximal part and measure the reduction. Then, the main stem (distal fitting type with normal size) is utilized. After reduction, stability of the hip joint is assessed and limb length discrepancy is checked.

Physiotherapy was started the day after surgery. All patients were followed up for at least 12 months, and Harris Hip Score questionnaire was applied to study the outcome; the patients were asked to fill in the questionnaire before surgery. During the follow-up visits, it was filled in once more after 12 months post-surgery.

## Result

A total of 42 patients with unilateral and 5 with bilateral DDH were studied (52 hips). Mean age of patients was 41 years (range 19–55 years). The minimum follow-up ranged from 12 months to 3 years. Two patients were lost to follow-up between 9 and 12 months. Uncoverage of > 30% was observed in 10 hips for which structural bone grafting was performed. Transverse subtrochanteric osteotomy was also performed in 24 hips.

Mean Harris Hip Score preoperatively was 41.70 (range 32–46) and postoperatively at last follow-up was 88.1 (range 74–94). According to Harris Hip Score, postoperative pain and limping were reported to be absent or slight among the patients. Support was not needed in any cases, and the distance patients walked, use of public transportation, going up from stairs, sitting and putting on shoes and socks were near normal. Flexion contracture under 30°, fixed abduction under 10° and fixed internal rotation in extension under 10° were not observed in any cases. We observed leg length discrepancy of less than 2 cm; three cases had limb length discrepancy of about 2 cm that was well tolerated using shoe lifts. All the patients reported to have proper range of motion and were satisfied with the outcome of surgeries. Clinical improvement in all patients was observed; all patients had the ability to walk normally without any help at 3 months after surgery (videos 1 and 2).

**Fig. 7:**
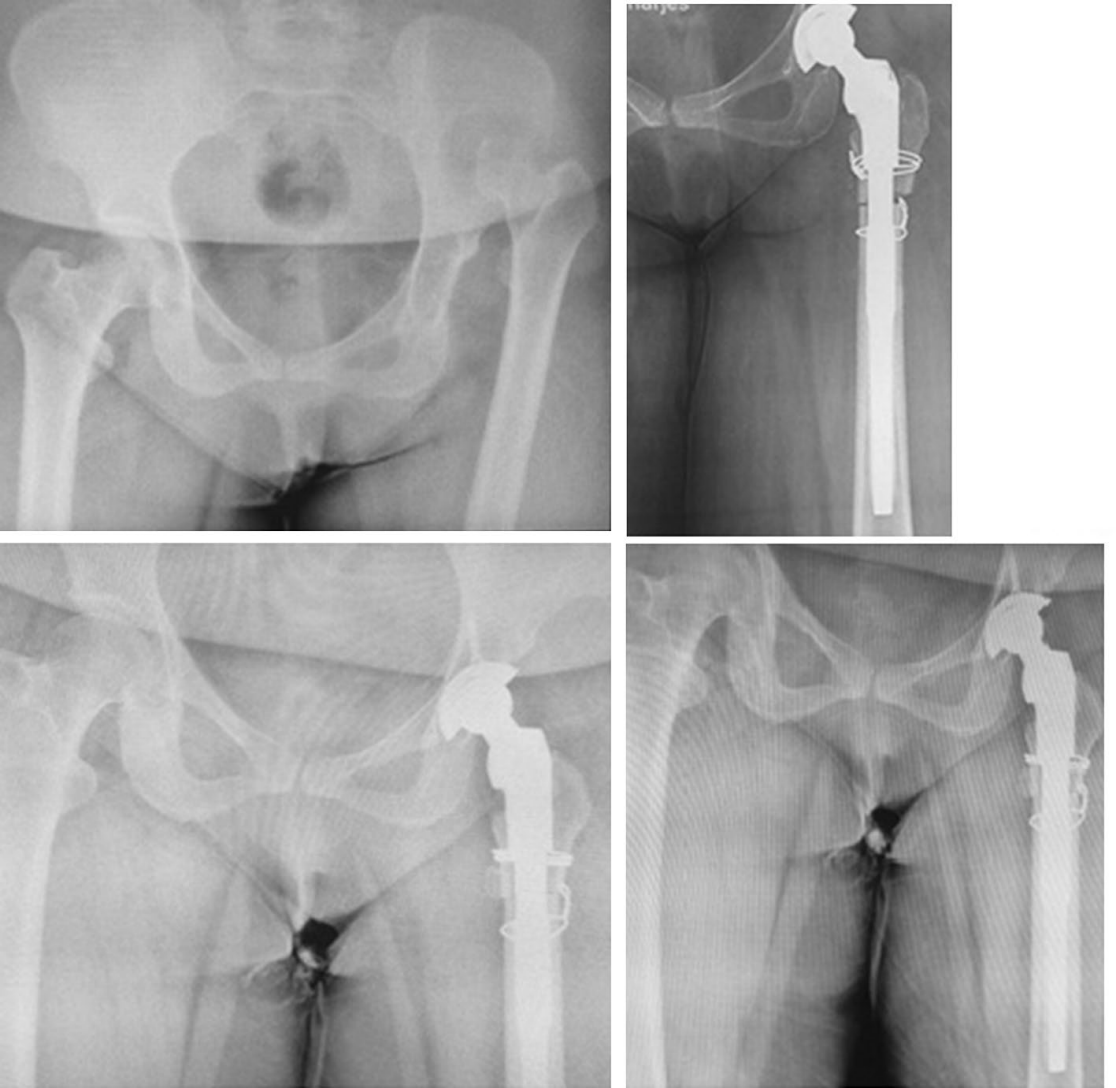
High-riding DDH undergone THR with cup placement in true acetabulum and transverse subtrochanteric osteotomy. Follow-up radiographs at 12th and 18th months reveal union of osteotomy site

Considering the postoperative complications, two patients had transient peroneal nerve palsy in early postoperative period which recovered within 2 months. No early or late wound dehiscence of infection occurred. No patient had iatrogenic vascular injury to femoral artery. We also did not encounter any iatrogenic fracture at the proximal femur. One case reported to suffer from falling 15 months after surgery who successfully underwent revision. No nonunion or heterotopic bone formation was seen in osteotomy site (Fig. 7). The mean leg length discrepancy was 5 cm preoperatively (range 2–7 cm) and 1 cm postoperatively (range 0–2 cm). We did not observe any septic or aseptic loosening of the components.

## Discussion

THR in Crowe type III and IV DDH is a challenging surgery. The most important goals are restoration of near-normal biomechanics and achieving sufficient cup coverage. Dysplastic hips have one single pathophysiological feature in common in which anatomic abnormalities intensify contact stress leading to degenerative arthritis [4]. Biomechanics and anatomy are altered with hypoplastic true acetabulum, excessive femoral anteversion, narrow medullary canal, proximal migration of femoral head and defective abductor mechanism [1–6].

Even though there are varieties of alternatives to non-arthroplasty treatment of DDH, there are a remarkable number of patients reported to have received total hip replacement as the treatment. Due to some certain characteristics of patients such as young age as well as anatomic abnormalities of hip, THR failure rate and complications in DDH patients are higher than normal population [9, 10]. Shallow socket with bone deficiency at anterior, lateral and superior areas of acetabulum is considered as one of the vital changes. THR, particularly determination of implant spot for acetabular component, causes new center of hip rotation which consequentially affects hip biomechanics, leg length and femoral reconstruction. Due to anatomic abnormalities, the use of standard-sized cup in dysplastic acetabulum leaves parts of component to be uncovered from native bone. A wide range of prostheses should be available, to choose the appropriate one for such hips. Absence of support intensifies the stress in the bone–implant or bone–cement interface which eventually leads to mechanical failure. The native bone should cover 70% of component surface as to generate the sufficient sustainability which allows for the bone ingrowth [11].

Once the sufficient coverage of the main bone is not attained by an implant, an alternative method needs to be applied. Two most important issues to be considered in these patients are the site of acetabular cup placement and fixation method. High rate of mechanical failure with cemented acetabular components without structural augmentation has been reported. A failure rate of 16–25% over a follow-up period of 10–20 years has also been reported. Younger ages during surgery, intense dysplasia with displacement of femoral head toward proximal and unanatomic acetabular component all result in weaker prognosis [11–13]. This demonstrates that when cemented acetabular component is used and cup coverage is required, no cup cement augmentation should be done. Over 10–12 years, about 40% of autograft-supported cemented acetabular component revealed signs of loosening of which 10–20% underwent revision [11, 14].

Lee et al. [15] stated that failure rate of 36 cases of cemented cup varied from 6% over 5 years to 39% in 10 years. Results came out to be far superior when hip center repositioned to its anatomic position with graft supporting less than 30–40% favored with superior and posterior wall. Kobayashi and colleagues [16] reported that there was no evidence of clinical failure when it came to cemented cups for 19 years. Results are much better once the graft supports less than 30–40% of component and a good job of superior support as posterior support is done. Huge bone grafts which interact with cement sockets do not typically have optimal longevity. Yet if reconstruction fails, bone graft takes much of bone stock and facilitates revision surgery.

There are plenty of techniques when it comes to non-augmented cemented acetabular component. Anderson and Harris [17] reported 20 cases of dysplastic hip reconstructed with non-cemented hemispheric cup followed up for about 6.9 years. Native bone covered 7–100% of the socket with component settled up to 28 mm proximal from inter-teardrop (5–66 spectrum) (some were high hip centers). None of sockets underwent revision surgery with no sign of loosening, migration or complete radiolucent line. This all attests to the vitality of non-cemented reconstruction in dysplastic hips.

Positioning acetabular component higher in proximal section for patients whose acetabular component in true acetabulum requires graft for provision of component support is recommended by Russoti and Harris [18]. Embedding of acetabular component in proximal section suits the elderly patients where anatomic position of acetabulum leaves more than 40–50% of socket surface uncovered and requires bone graft. Pagnano et al. [19] on the contrary found high rate of loosening of components with proximal position. Russoti and Harris [18] and some other authors favored more proximal but not lateral placement of acetabular component. Doehring et al. [20] found that superior only placement may be mechanically acceptable.

**Fig. 8:**
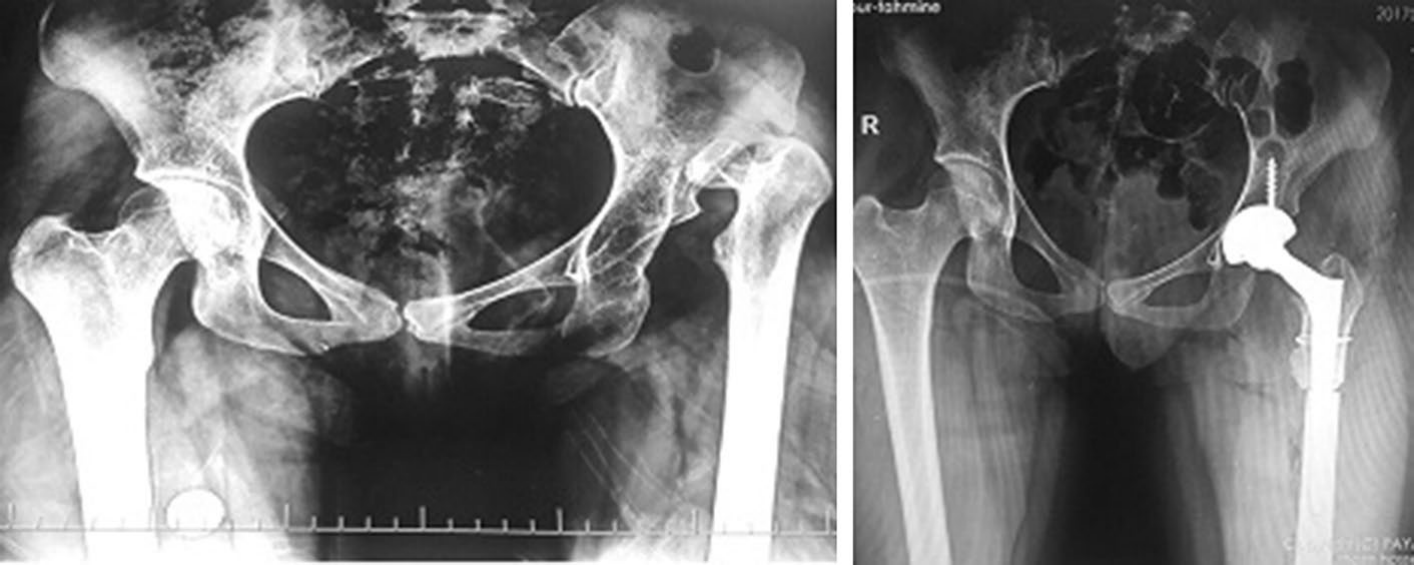
Another case of high-riding DDH undergone THR with cup placement in true acetabulum and transverse subtrochanteric osteotomy

**Fig. 9:**
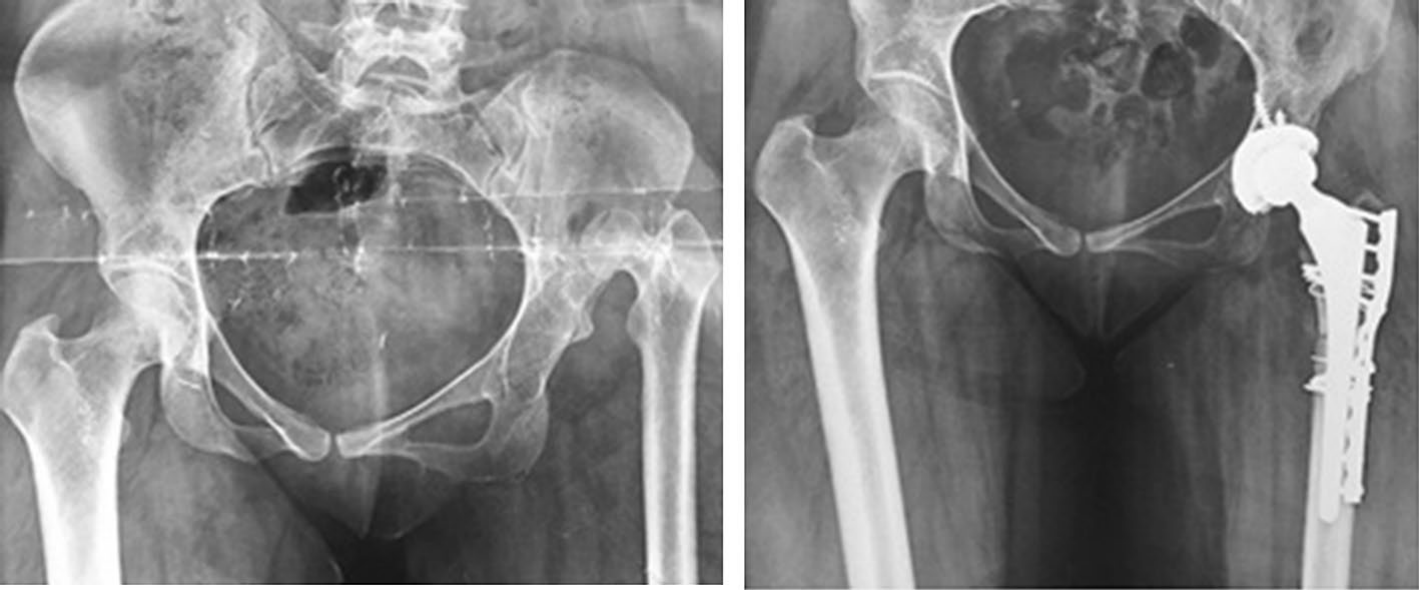
A high-riding DDH undergone THR with cup placement in true acetabulum and cotyloplasty; transverse subtrochanteric osteotomy was done and fixed with a plate

According to the available articles, there are no documented data on the prevalence of neglected DDH in Iran. However, many cases of infantile DDH were unfortunately neglected in the very past in the rural area of Iran with low socioeconomic living conditions; as a result, adult-type high-grade DDH is not uncommon in such areas. As a tertiary referral center, we are having 3–5 new patients with neglected DDH visited at our clinic mostly from western areas of Iran every 2 weeks. Here in this article, we present our experience with DDH patients who underwent reconstruction of acetabulum at true acetabulum level (Figs. 8, 9). Placement of acetabular component in true acetabulum level restores a near-normal biomechanics while providing the best available bone stock for cup placement [3, 21] making it the ideal place for acetabular cup placement [22, 23]. When femoral head is proximally dislocated, the iliac bone stock becomes very deficient and securing the cup at or near true acetabulum becomes a challenge. For enhancement of the cup coverage, we used medialization of the acetabulum with small-sized cup and structural bone grafting. Most authors accept that if coverage is between 60 and 80%, augmentation should be used and coverage with graft should not be more than 30–40%. In our study, 10 hips had an uncoverage of > 30% for which structural bone grafting was performed. By use of a smaller component, good resection of medial osteophyte and adequate medialization at cotyloid fossa, we were able to place acetabular cup at true acetabulum.

In our study, transverse subtrochanteric osteotomy was done in 24 hips. Many authors reported good result with low rate of nonunion of osteotomy site [24–27]. We favor to use uncemented press-fit distally fixed stems. Many authors reported excellent survival with modular uncemented stem [9, 10, 23, 24].

## Conclusion

Performing THR in patients with DDH (Crowe III and IV) with a cup positioned in true acetabulum and transverse subtrochanteric osteotomy is a safe successful procedure. Small-sized cup with medialization of acetabulum produces good results. We favor uncemented press-fit acetabular cup and modular uncemented stem with transverse subtrochanteric osteotomy. However, there is no universally accepted standard treatment for Crowe III and IV dysplastic hip and each case needs individualized treatment.

## Electronic supplementary material

Below is the link to the electronic supplementary material.

Supplementary material 1 (MP4 971 kb)

Supplementary material 2 (MP4 1360 kb)
